# The Impact of Molecular Weight Distribution on the Crystalline Texture of Polymers—Illustrated by Lamellar Crystal, Shish–Kebab and Nested Spherulites

**DOI:** 10.3390/ma18174196

**Published:** 2025-09-07

**Authors:** Songyan Lu, Min Chen, Hanying Li

**Affiliations:** MOE Key Laboratory of Macromolecular Synthesis and Functionalization, International Research Center for X Polymers, ZJU-YST Joint Research Center for Fundamental Science, Department of Polymer Science and Engineering, Zhejiang University, Hangzhou 310027, China; lusongyan@zju.edu.cn

**Keywords:** polymer crystallization, molecular weight distribution, molecular segregation

## Abstract

Manipulating polymer crystallization behavior and structure without altering chemical composition remains a core challenge in polymer crystallography. Molecular weight, as an intrinsic material property, governs the crystallization process from nucleation through growth. Synthetic polymer materials exhibit molecular weight distribution (MWD), resulting in polydisperse polymer chains within one system. This MWD drives distinct crystalline structures, whereas synergistic crystallization behaviors arise among chains of various lengths. MWD yields complex crystallization behaviors. Especially, spatial molecular weight distribution induces novel crystalline textures in polymer materials. Elucidating the crystallization mechanisms is vital for understanding structure-property relationships in polymers. Herein, the recent advances in the various influences of MWD on polymer crystal textures are systematically demonstrated.

## 1. Introduction

Synthetic polymer materials typically exhibit a molecular weight distribution (MWD), meaning that one polymer system coexists with polymer chains of various lengths. Molecular weight(MW) plays a critical role in polymer processability and physical properties [1,2,3,4,5], through its governing effects on crystallization kinetics, including both nucleation and growth processes [6,7,8,9]. Fundamentally, MW governs polymer properties by directing aggregated structure formation [10,11,12], which manifests through chain entanglement (e.g., entanglement density [13,14,15]) and crystallization behaviors (including lamella thickness [9,16,17,18] and crystal morphology [19,20,21,22,23]). The phenomenon that polymers can form complex crystalline structures under identical crystallization conditions has attracted researchers’ attention, exemplified by polymorphism during isothermal crystallization in polydisperse polymer. MWD may be the underlying cause for this phenomenon, which implies that chains of multiple lengths crystallize simultaneously [24,25,26,27,28,29,30]. The macroscopic properties of polymer materials, such as mechanical properties [31,32,33], thermal stability [18,20,34], and electrical properties [23,31,35,36,37], are determined by crystalline structure, which is decisively influenced by MWD. The significant effect of MWD on crystallization kinetics, crystallinity, and the final crystalline textures is sparking widespread research interest.

MW effects on crystallization remain a core research subject. Understanding of these effects has shifted from interpreting MWD as a single parameter to recognizing the distinct contributions of various molecular weight fractions within the MWD curve [8,38,39,40]. Different molecular weight components in polymer materials are not entirely uniformly mixed. Instead, they may be spatially distributed or express distinct crystallization behaviors in the same polymer material. The breadth and shape of the MWD curve determine the proportion of high molecular weight (HMW) and low molecular weight (LMW) components within the system and their combined crystallization behaviors. The effect of MWD on polymer crystallization has been widely studied in the crystallization kinetics of linear unimodal and bimodal polymers [41,42,43,44,45]. One of the general understandings of the molecular weight effect on crystallization is that HMW components exhibit high entanglement density and slow relaxation kinetics; conversely, LMW components possess high chain segment mobility. Further revealing the effects requires directly measuring the relationship between MW and crystallization behavior in crystallized polymers, but this remains challenging. Given this limitation, researchers have attempted to establish correlations with MW and varying crystalline structures within polydisperse polymer systems, thereby inferring crystallization behavior of different MW components [6,41,43], from basic crystal morphologies (e.g., lamellae [9,46,47,48,49,50,51], spherulites [52,53]) to crystalline textures (e.g., shish kebab [25,26], patterned single crystals [17,30,54,55], spherulitic textures [27,28,29]). The specific influence of MWD on crystalline structure is multifaceted, with the distinct roles of HMW and LMW components in determining nucleation and growth steps under flow fields [25,26,34,56], and the propensity for different crystal polymorphs [17,22,57,58,59] to form in HMW versus LMW components under identical crystallization conditions.

Therefore, systematically deciphering the multiscale effect of MWD on polymer crystallization behaviors and crystalline textures holds not only theoretical importance but also guides the molecular design of high-performance polymers and optimizes processing techniques. This review selects several representative works as examples, such as the impact of MWD on crystalline textures like shish kebab and nested spherulites. It introduces the effects of MWD on polymer crystallization behaviors and textures, discusses the underlying mechanisms, and particularly incorporates the perspective of molecular weight spatial distribution (MWSD) to discuss this issue.

## 2. Impact on Crystalline Structures Rooted in Molecular Segregation

Researchers have long recognized the impact of chain length on crystallization behavior. The Lauritzen−Hoffman model stands as the most widely adopted model for studying MWD and temperature effects [60,61]. It states that crystal growth is controlled by both chain transport and secondary nucleation at the lateral growth front of lamellar polymer crystals, and assumes that the deposition of the first stem overcomes the free energy barrier of secondary nucleation. Mandelkern and coworkers established that crystallinity, melting temperature, and crystalline morphology in isothermally crystallized polydisperse polyethylene exhibit pronounced MW dependence [62,63,64,65,66,67,68]. Their results demonstrate that during isothermal crystallization of polyethylene prepared by rapid quenching/precipitation from dilute xylene solution, LMW components (below 5000) do not co-crystallize, while those with MW of 10,000 or higher participate in the crystallization process. Industrial polymerization conditions inherently yield polymer materials with MWD. Consequently, the crystallization behavior of semicrystalline polymers frequently involves the cooperative influence of multiple MW components. Thus, the impact of MWD on crystalline structure is an inherent consideration in practical polymer crystallization.

**Figure 1:**
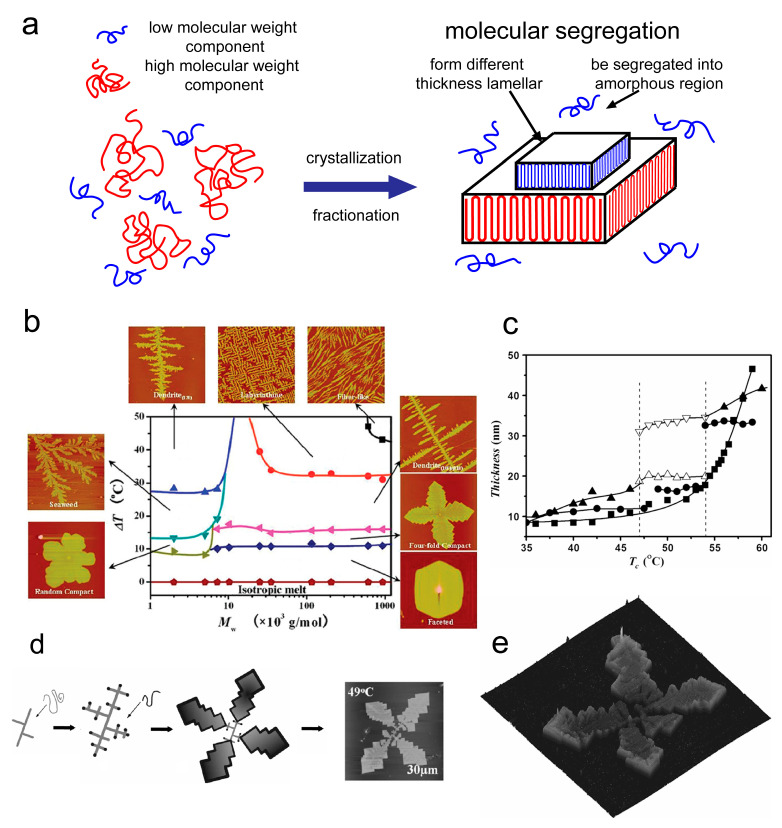
Schematic diagram of the molecular segregation phenomenon and distinct crystalline patterns in polymers arising from molecular segregation. (**a**) Schematic diagram of molecular segregation. (**b**) Morphology diagram of the PEO single-layer crystal patterns, the areas between different curves represent the growth patterns formed within specific MW and temperature ranges, which correspond to the images as arrow pointed [30]. Reproduced with permission, copyright 2012, American Chemical Society. (**c**) The average thickness of the crystalline textures corresponding to 35k-PEO (■), 5k-PEO (●), and their blend (▲). ▽ is the edge thickness of the blend, and △ is the center thickness of the blend. (**d**) Schematic illustration showing the effect of molecular segregation on crystal pattern and its AFM image. (**e**) 3D AFM image of the PEO blend, which crystallizes at 49.0 °C [17]. Reproduced with permission, copyright 2009, Elsevier Ltd.

The molecular segregation during the crystallization process is the fundamental mechanism on which crystallization fractionation is based. In polydisperse polymers, molecular segregation is manifested by the separation of MW components into distinct fractions during crystallization (as shown in Figure 1a). Richards demonstrated that the HMW fraction of branched polyethylene crystallizes preferentially at elevated temperatures in solutions [69]. Numerous experimental studies have established this molecular segregation during polymer crystallization [70,71,72]. A classic manifestation demonstrates that polyethylene fractions with narrower MWDs could be collected through crystallization and filtration at different temperatures [73].

Substantial research explores the relationship between molecular segregation driven by MWD and crystallization. It poses a challenge to polymer crystal growth models, such as clarifying the nucleation and growth process. Hoffman proposed that besides the secondary nucleation barrier for the first stem lying on the smooth growth front, there exists an additional barrier associated with MW for each new polymer chain reeling in the crystal [74,75]. This additional barrier represents the energy required for a polymer chain to disentangle from the melt and be reeled in to the crystal growth front. Through selective dissolution of polyethylene after isothermal crystallization from the melt under high temperature and pressure, Mehta and Wunderlich successfully demonstrated that molecular segregation takes place during isothermal crystallization. They posit that only sufficiently long polymer chains can achieve the requisite number of chain folds to attain the stable size for a nucleus at the crystal growth front [76,77]. In order to consider the multiple nucleation cases of a single chain, Hu et al. contended that secondary nucleation is dominated by intramolecular nucleation events involving chains of various lengths through Monte Carlo simulation calculations. Molecular segregation during polymer crystal growth and the observed upper MW limit are both compellingly explained by the intramolecular crystal nucleation model [11,12].

Molecular segregation profoundly influences polymer crystallization structures based on MWD. In polymer blends with MWDs, the presence of distinct MW components leads to the formation of different crystalline structures (as shown in Figure 1b) [30]. For instance, in blends of two poly(ethylene oxide) (PEO) fractions, partial segregation occurs within PEO co-crystallizations over temperature ranges. The MW corresponding to positions in the crystal textures was obtained by measuring the average crystal thickness of single MW component and their blend (as shown in Figure 1c). As shown in Figure 1d,e, it results in crystalline textures comprising thin-lamellar dendrites in the interior, surrounded by thicker lamellae at the periphery [17,30,54]. This research suggests that the HMW component (e.g., 35k-PEO) nucleates first, forming lamellae with non-integer fold chains. (A non-integer fold chain is a molecule chain that does not fold back on itself an integer number of times, with its chain ends inside crystalline regions or in the amorphous regions, leaving dangling segments or forming tie molecules.) The crystal edges extended-chain lamellae formed by the LMW component (e.g., 5k-PEO). Consequently, a spatial distribution of MW emerges from molecular segregation, and this spatially varying MWD gives rise to a composite crystalline texture featuring distinct lamellar structures and thicknesses in the interior and periphery.

**Figure 2:**
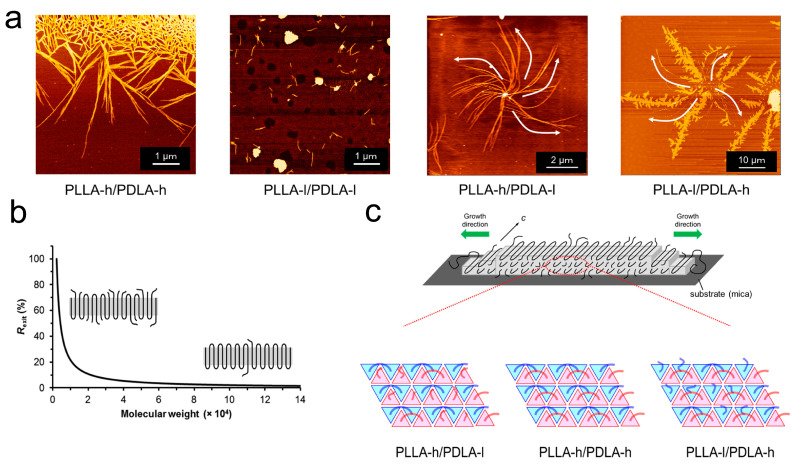
The crystal structures and formation mechanism of PLLA/PDLA stereocomplexes blended with multiple MWs. (**a**) The AFM images of the PLLA/PDLA thin films with equivalent or nonequivalent MW. (**b**) The curve of MW versus the ratio of PLA chains exiting from the lamella without folding. Schematic illustration represents the different molecular chain folding states in varying MW. (**c**) Top schemes of the edge-on crystal of the PLLA/PDLA stereocomplex. Bottom schemes show a different ratio of chain folding for PLLA and PDLA chains in the edge-on stereocomplex crystals across various combinations of MW [24]. Reproduced with permission, copyright 2013, American Chemical Society.

The influence of MWD on crystal structure is also reflected in poly(L-lactide) (PLLA)/poly(D-lactide) (PDLA) stereocomplexes with equal mass but different MW [24]. Researchers blended HMW poly(L-lactide) (PLLA-h, PDLA-h) and LMW PLA (PLLA-l, PDLA-l) components [24]. It was found that when PLLA and PDLA of differing MW are blended, the edge-on crystals of the PLLA-h/PDLA-l stereocomplex curved predominantly to the left as a Z-shape. The PLLA-l/PDLA-h crystal structure curved predominantly to the right in an S-shape. In addition, in PLA stereocomplexes where PLLA and PDLA components had equivalent MW (e.g., PLLA-h/PDLA-h and PLLA-l/PDLA-l), edge-on lamellae grew linearly (Figure 2a). Crucially, the direction of crystal curvature is consistently dictated by the chirality of the low-molecular-weight component. The researchers attribute this to the ratio of chains exiting the lamella surface without folding, which is MW dependent (Figure 2b). As MW decreases, polymer chains find it progressively more difficult to fold under identical crystallization conditions, ultimately adopting extended chains within the crystal. The LMW components of both chiral polymers (PLLA and PDLA) within the stereocomplex exhibit higher ratios of chains exiting without folding (Figure 2c). The influence of the LMW component on the ratios of unfolding chains governs the surface stress on the lamellar crystal, which results in curving and twisting of crystalline lamellae.

## 3. Molecular Weight Distribution Effect on Shish Kebab Structures Under Flow Fields

Polymer processing conditions demonstrably alter crystalline morphology and the rate of solidification, ultimately governing material properties. The application of flow exerts a profound influence on the crystalline structure in polymer processing. Flow-induced perturbation of chain conformation critically affects polymer crystallization behavior, driving extensive research into shear-induced crystallization of polymers. Shish kebab is a typical and frequently observed polymer crystalline morphology formed under shear fields. This crystal structure comprises an oriented central fiber core (shish) overlaid with epitaxially grown lamellae with a growth direction perpendicular to the shish (kebab).

Notably, MWD plays a decisive role in the formation of unique crystalline structures, such as the shish kebab induced by flow fields. The abrupt coil-stretch transition model is one of the theoretical frameworks for the formation mechanism of shish structures in early stages. This model proposes that polymer chains in dilute solution undergo an abrupt transition from a random coil conformation to a fully extended chain conformation at a critical extensional strain rate. The transition of polymer chains occurs without passing through any stable intermediate states. Considering entanglements among molecular chains, Keller and coworkers proposed that the longest chains are the primary driver for forming stable shish [78,79,80]. Their long relaxation times favor resistance to disentanglement under flow. HMW components extension along the stress direction facilitates the formation of shish structures. Conversely, LMW components dominate the rapid growth of kebabs due to their higher diffusion rates. LMW component chains crystallize as chain-folded lamellae at nucleation sites on the shish, enabling subsequent radial growth. Simulations indicate that long chains stretch to form a shish kebab core, around which short chains aggregate into a kebab structure [79].

Simulation works indicate the formation of oriented shish by long chains with peripheral kebab structures of short chains. Furthermore, recent experimental studies have observed the distribution of HMW and LMW components within the shish kebab structures. Counterintuitive MW dependence was observed in the formation of shish kebab structures within linear low-density polyethylene (LLDPE) and HMW/LMW high-density polyethylene (HDPE) blends [56]. The two-dimensional wide-angle x-ray scattering (2D-WAXS) patterns of blend samples exhibit significant orientation degrees. Furthermore, the blend containing LMW HDPE displays significantly higher orientation than its HMW HDPE counterpart. Contrary to expectations based on the longer relaxation time of HMW HDPE, which should yield higher orientation, the observed orientation of blends with HMW HDPE was actually lower. These results indicate that incorporating LMW HDPE more effectively enhances crystalline orientation under shear. Compared to HMW HDPE, the higher chain mobility and lower entanglement density of LMW HDPE enable it to produce a more highly anisotropic shish kebab structure in oriented LLDPE/HDPE blends subjected to oscillatory shear during injection molding packing. That study found that chain mobility plays a critical role in the microstructure of crystals after shear-induced thin film formation. The impact of LMW components on crystal structure is manifested as an increased degree of orientation, more compact layered lamella stacking, thicker thin films, and higher melting temperatures.

**Figure 3:**
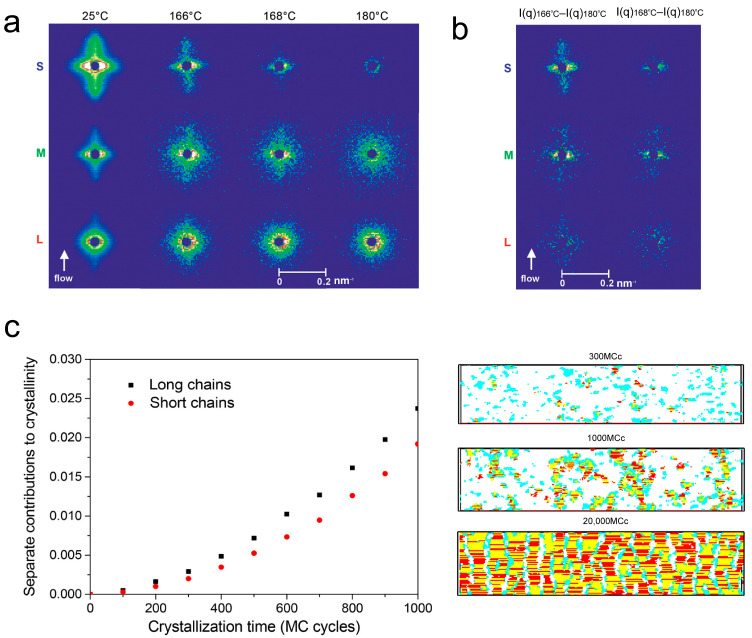
New insights into the spatial distribution of HMW and LMW in the shish kebab. (**a**) SANS profiles of deuterium-labeled materials during heating from 25 °C to 180 °C. S, M, and L stand for the model i-PP, which labels chains in the shortest third, the middle third, or the longest third. (**b**) The difference in SANS scattering intensity between 166 °C and 180 °C (left) and between 168 °C and 180 °C (right) for S, M, and L [25]. Reproduced with permission, copyright 2007, The American Association for the Advancement of Science. (**c**) The curves of crystallization time versus crystallinity contributions separately from the long and short chains during isothermal crystallization (left) and snapshots of precursors and crystallites at 300, 1000, and 20,000 Monte Carlo cycles (right) [80]. Reproduced with permission, copyright 2018, American Chemical Society.

Through deuterium labeling chains of varying lengths combined with Small-Angle Neutron Scattering (SANS) experiments, Kimata et al. revealed that long chains are not overrepresented within the shish relative to their overall concentration in the material [25]. Specifically, SANS patterns from three model isotactic polypropylene (i-PP) resins containing labeled chains in the shortest, middle, or longest third of the MWD showed that the chain length distribution within the shish core closely resembles that of the bulk melt (Figure 3a). In addition, this study excluded the interference caused by the relatively strong melt scattering of long-chain melts by comparing the signal differences during the heating process (Figure 3b). Critically, the experiments confirmed that the presence of long chains is essential for enhancing the propagation of shish structures. The shish structure forms rapidly because long chains markedly accelerate shish propagation by achieving high degrees of segmental orientation upon adsorbing to the tip of a growing shish. Without long chains, effective shish formation is impeded. As shown in Figure 3c, the work of Nie et al., validated through dynamic Monte Carlo simulation, may explain Kimata et al.’s observation. In the early crystallization stage, LMW and HMW components participate in the formation of the highly oriented thin-layer structure, which is considered the precursor for the formation of shish in the shear flow. The HMW components, as the deformed subchains, directly determine the formation of crystalline bonds. The extent of LMW components’ involvement was limited by the chain segregation [80]. From a MWSD viewpoint, HMW and LMW components exert distinct crystallization speed within the shish and kebab domains; the HMW components exhibit a faster crystallization rate of crystal growth during the formation of the shish, especially.

**Figure 4:**
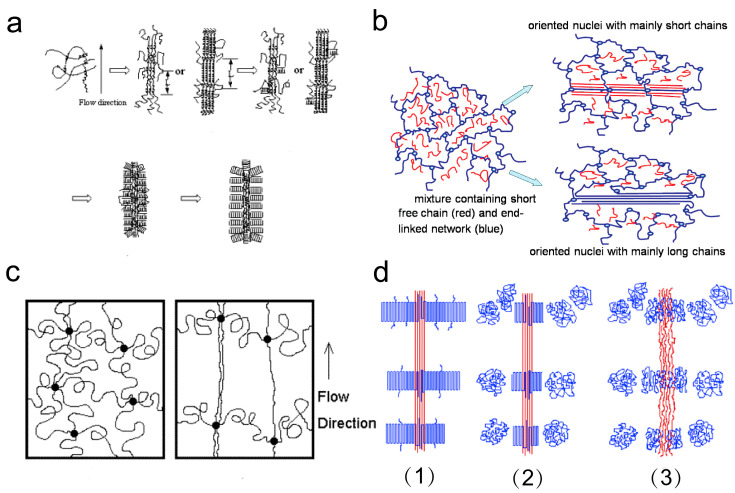
The formation models of shish kebab structures. (**a**) Schematic illustration of the morphological development during shear-induced crystallization i-PP from the stretched network model [82]. Reproduced with permission, copyright 2005, Elsevier Ltd. (**b**) Flow-induced crystallization model: the oriented nuclei with mainly short chains in melt (top) or disentangled long chains in polymer solutions (bottom) [83]. Reproduced with permission, copyright 2009, American Chemical Society. (**c**) Schematic illustration of the entanglement of the HMWPE chain network. Under the uniaxial shear field, the segments stretch along the flow direction, and most of the segments remained in the coiled state as left. The stretched segments form precursors for shish formation, while the coiled segments further grow into kebabs. (**d**) Schematic representation of the shish kebab structure at three stages: (1) stable shish kebab structure after isothermal crystallization, (2) the melting of macro-kebabs during heating, (3) the melting of micro-kebabs and shish at a higher temperature [34]. Reproduced with permission, copyright 2006, American Chemical Society.

Recent research further revealed the impact of HMW and LMW fractions on shish kebab formation during the crystallization process [81]. A broader MWD favors shish formation: it slightly reduces the induction time during the first stage of the emergence of shish precursors, and significantly shortens it in the secondary stage of evolution from precursors to shish nuclei. This promotes both the emergence of shish precursors and evolution into stable nuclei, while accelerating the overall crystallization rate of the shish kebab structure. Broadening the MWD, particularly by increasing the HMW fraction, leads to more regular and dense tie molecules within the crystal structures, and this process triggers lamellae thickening. With recent research, Zhang et al. have revealed that i-PP undergoes disentanglement and forms shish kebab structures even at shear rates significantly below the Newtonian-to-non-Newtonian transition threshold. Under a shear field, polymer chains experience network stretching, alignment, and enhancement of helical segments or clusters. Ultimately, these chains organize into a crystalline structure with a correlation length corresponding to the average MW between entanglement points in i-PP melt (as illustrated in Figure 4a) [82]. Zhao et al. proposed that whether the entanglement-disentanglement(EDT) phenomenon occurs in blends of minor long chains and major short chains affects the MW composition of the shish within polydisperse PEO blends. In polymer melts during processing, where the EDT rarely occurs, flow-induced oriented nuclei are primarily composed of LMW components. Conversely, in polymer solutions where EDT is present, the oriented nuclei are mainly formed by disentangled HMW components (as shown in Figure 4b) [81]. Concurrently, Zuo et al. focused on the formation and thermal stability of shish kebab in a polymer blend containing 98 wt% LMW PE and 2 wt% narrowly distributed crystallizing HMW PE. This research suggested stretched and coiled chains coexist in near monodispersed HMW components under a shear flow field. The highly entangled HMW components melt, with each entanglement point acting as a physical cross-link. The melting behaviors of shish and kebab also differ. The melting of the shish kebab structure occurs in two stages. The kebabs first melt from structurally well-defined macrokebabs into microkebabs, while the shish remains unmelted at this stage. In the next step, the microkebabs and the shish melt simultaneously, transforming the coiled and stretched chain segments as an integrated entity (as shown in Figure 4c,d) [34].

## 4. Role of Molecular Weight Distribution in Solution-Processed Polymer Spherulite Films

**Figure 5:**
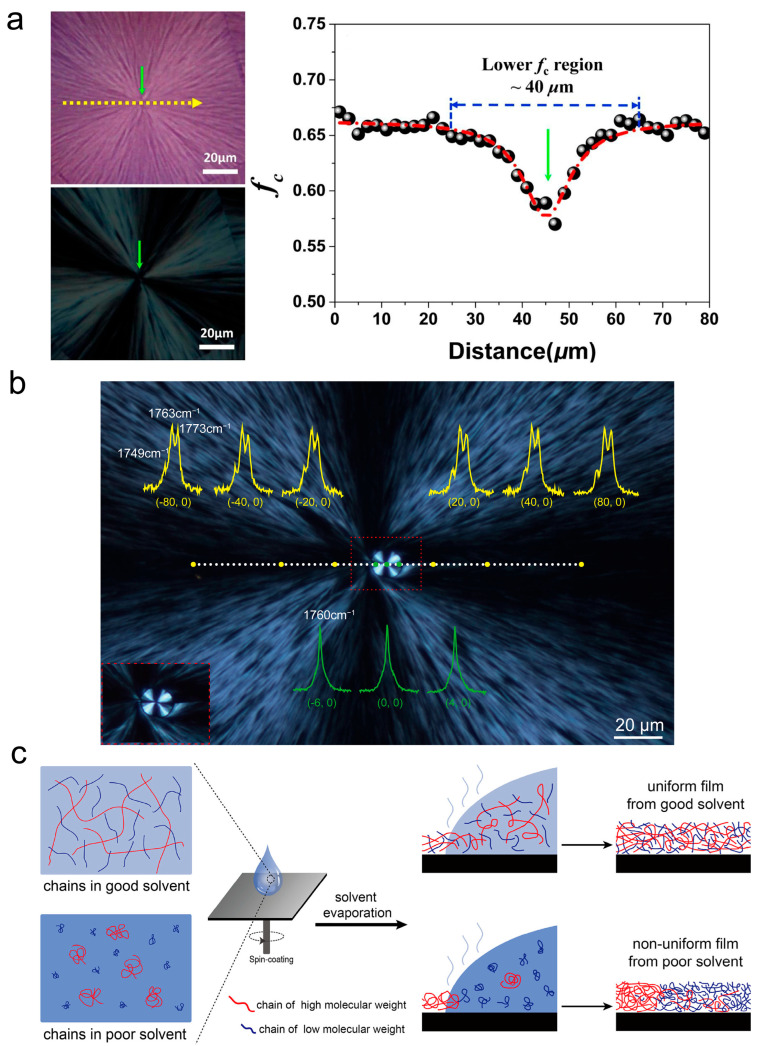
Novel crystalline textures in polymer spherulites and MW-dependent formation mechanisms. (**a**) Line scanning Raman measurement of PCL spherulites. The profiles of the proportion of the integral intensity for the crystalline component along the scanning direction [84]. Reproduced with permission, copyright 2018, Elsevier Ltd. (**b**) Polarizing optical microscopy (POM) image of the PLLA nested spherulites with representative Raman spectra from the central α’-form spherulite (green dots) and the external larger α-form spherulite (yellow dots). (**c**) Schematic illustration of MWSD heterogeneity in PLLA arising from fractional precipitation during solution spin-coating [28]. Reproduced with permission, copyright 2022, American Chemical Society.

Spherulite is the prevalent crystalline morphology, which can be found in a wide range of semicrystalline polymers. The MWD directly influences structural features such as radial crystallinity gradients from nucleation sites and the formation of specific crystal textures. Yang et al. identified and separated the characteristic signals from crystalline and amorphous regions in poly(ε-caprolactone) (PCL) semicrystalline films by using the Raman spectrum. The Raman microscopy enables spatial mapping of crystallinity distribution within PCL spherulites [84]. Their study revealed lower crystallinity at spherulite nucleation sites versus significantly enhanced crystallinity at spherulite peripheral regions (as shown in Figure 5a). This graded crystalline texture is attributed to molecular segregation during polymer crystallization.

The impact of MWD on spherulite texture would stem from fractional precipitation during solution spin-coating. In polydisperse polymer systems, differing solubility causes HMW components to precipitate first under identical conditions, forming spatially segregated HMW aggregates, while LMW components subsequently form distinct LMW aggregates. This spatial MW segregation enables HMW and LMW fractions to exhibit distinct crystallization behaviors under equivalent crystalline conditions. An example is the nested spherulite structure observed in PLLA (as shown in Figure 5b) [27,28]. At the same crystallization temperature, HMW components PLLA aggregates preferentially form less thermally stable α’-form spherulites. It is due to the higher entanglement density of HMW hindering disentanglement and reorganization into ordered structures. In contrast, LMW components adopt the thermodynamically stable α-form during crystallization. Nucleation within HMW aggregates initiates α’-form spherulites; subsequently, faster-growing α-form spherulites nucleate at boundaries of α’-form spherulites and embed them, forming the characteristic nested spherulites crystal texture. The formation of nested spherulites demonstrates that the presence of MWD during solution processing enables simultaneous crystallization of multiple crystal forms under isothermal conditions.

To resolve position-dependent crystalline structural variations in polymer crystalline films, particularly polymorphism, this study employed Raman spectroscopy mapping with 10 μm spatial resolution and non-destructively acquired crystal form distribution data [27,28]. The experimental observation focused on a polydisperse PLLA film prepared by solution spin-coating, which exhibited the nested spherulites structure of α-form and α′-form crystals after isothermal crystallization. Furthermore, leveraging differential solubility between α-form and α’-form crystals in PLLA under the same solvent and temperature conditions, selective solvent etching was implemented to isolate the MW components constituting distinct polymorphic spherulites, with subsequent MW characterization. The experiment ultimately separated PLLA crystalline materials from distinct spatial regions, and Gel Permeation Chromatography (GPC)measurements confirmed that the α-form and α’-form crystals are composed of LMW and HMW components of PLLA, respectively. This methodology experimentally verifies spatial heterogeneity in MWD within polymer materials and demonstrates how such heterogeneity drives polymorphic differentiation under isothermal crystallization conditions.

**Figure 6:**
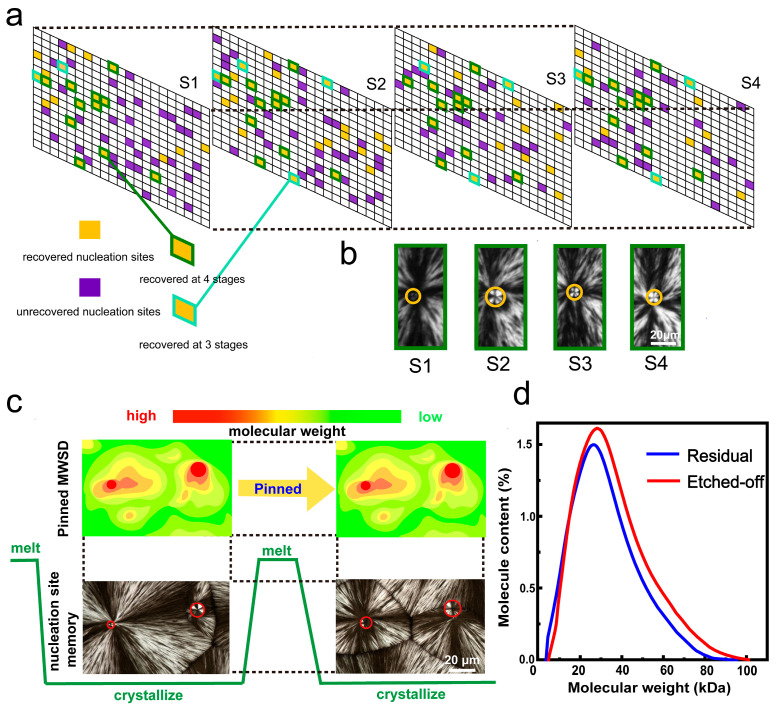
Nucleation site memory phenomenon in PLLA crystalline films and the possible MWSD formation mechanisms. (**a**) Schematic illustration for representing the positions of nucleation sites in a PLLA crystalline film in four stages, which shows the recoverability of nucleation sites for each stage. (**b**) POM images of recovered nucleation sites that recovered four stages. (**c**) Schematic illustration of the pinned-MWSD of PLLA films during the thermal cycles. (**d**) GPC curves of the etched-off PLLA component (red) and the residual (blue) [29]. Reproduced with permission, copyright 2024, American Chemical Society.

The essence of the polymer memory effect lies in residual ordered structures, accelerating nucleation, and recapitulating prior crystallization processes. This effect is primarily associated with the thermal history of the material in prior studies, as these residual ordered structures survive or originate from previous thermal processing. However, a distinct nucleation site memory phenomenon emerges in PLLA nested spherulites (as shown in Figure 6a,b) [29]. Across multiple melt-crystallization cycles, spherulite nucleation sites consistently recover at nearly identical spatial positions, demonstrating spontaneous positional anchoring. It is noteworthy that within this nested structure, the α’-form spherulites, which are less thermally stable and harder to maintain the original structure, exhibit superior nucleation site recoverability, and this nucleation site memory strongly correlates with the solution-processed nested spherulite structures. Research reveals that during PLLA solution spin-coating, HMW components preferentially precipitate, establishing a graded MWSD. Subsequently, during melting, high melt viscosity and chain entanglement pinned HMW and LMW components within their respective aggregated region, preserving the MWSD across the crystal process (as shown in Figure 6c). Finally, HMW aggregates preferentially nucleate α’-form again, forming the nucleation sites of nested spherulites in a similar region at the next crystallization stage. This nucleation site memory phenomenon in PLLA is governed by the MWSD (as shown in Figure 6d). Fractionation precipitation pins HMW and LMW components to distinct spatial domains, which exhibit differentiated nucleation abilities and polymorph selection during subsequent crystallizations. It finally causes spherulite nucleation sites to repeatedly emerge at identical locations. The nucleation site memory phenomenon demonstrates the influence of MWSD on polymer crystallization behaviors, including nucleation ability and growth tendency of crystal forms.

## 5. Summary and Outlook

Building upon established research regarding molecular weight (MW) influence on polymer crystallization behaviors and structures, increasing scholarly attention focuses on molecular weight distribution (MWD) effects. Current evidence indicates that coexisting MW components may either cooperatively participate in crystallization processes or segregate spatially to form independent crystalline textures within the same polymer system. This review summarizes current understanding of crystalline texture formation in polymers directed by MWD, including lamellar thickness, curved crystal structure in PLLA/PDLA stereocomplex, shish kebab structures, and nested spherulites.

Previous MWD studies typically assume homogeneous mixing of high- and low-molecular-weight (HMW/LMW) components collectively influencing crystallization. Recent research has revealed that under specific processing conditions (e.g., solvent evaporation, spin-coating), HMW and LMW components segregate into aggregates with spatial distribution, forming heterogeneous crystalline structures across different spatial regions. Remarkably, this spatial partitioning of MW enables nucleation site memory phenomena independent of residual crystalline structure. Investigating MWSD effects expands fundamental knowledge of polymer crystallization kinetics, such as disentangling behaviors of HMW and LMW components in shish kebab structures between solutions and melts, and their dominance in the shish. It provides mechanistic explanations for the formation of some special crystalline structures, such as the formation of nested spherulites.

In summary, MWD significantly influences the crystalline structures of polymers. From an academic perspective, HMW/LMW components exhibit distinct crystallization behaviors within polymer materials, leading to the formation of complex crystalline structures such as shish kebabs and nested spherulites. Studying the crystallization behavior of different MW components is essential for elucidating the formation mechanisms of polymer crystalline structures. From a performance optimization perspective, crystallization behavior directly affects material properties through MWD. Understanding the relationships between material properties and MWD offers guidance for polymer processing and performance advances.

Research concerning the influence of MWD on polymer crystalline structure still faces numerous unresolved challenges. At the microscopic level, researchers posit that MW affects the ordering state of polymer chains by influencing factors such as entanglement density and degree of orientation. However, the underlying crystallization mechanisms hypotheses are often derived indirectly from comparative experiments or simulations. Those indirect comparative experiments frequently involve the less precise separation of polymer components or require destructive methods that disrupt the original crystalline polymer material, as exemplified by techniques like dissolution-etching experiments. Developing experimental techniques capable of directly characterizing the relationship between MWD and entanglement or orientation is important.

Regulation of MWD, particularly MWSD, enables targeted formation of specific crystalline structures at predetermined locations, thereby programming polymer material properties. Achieving high-precision spatial control over MWD represents a promising pathway for patterning polymer crystalline textures. This approach may constitute the fundamental strategy for developing advanced crystalline polymer materials with spatially regulated functionality.

## Data Availability

No new data were created or analyzed in this study. Data sharing is not applicable to this article.
